# Case Report: Dorsal inlay labial mucosa graft urethroplasty for pediatric and adolescent long-segment urethral stricture after failed hypospadias repair

**DOI:** 10.3389/fped.2026.1918653

**Published:** 2026-08-10

**Authors:** Ilaria Buconi, Lorna Spagnol, Leonardo Crescentini, Giovanni Rollo, Letizia Corbi, Massimiliano Silveri

**Affiliations:** Andrological Surgery Unit, Bambino Gesù Children's Hospital IRCCS, Rome, Italy

**Keywords:** case report, dorsal inlay urethroplasty, failed hypospadias repair, lichen sclerosus (LS), pediatric urethral stricture

## Abstract

Long-segment urethral stricture represents a major and challenging complication after multiple failed hypospadias repairs. In pediatric and adolescent patients, reconstructive strategies for these complex cases remain non-standardized, and clinical evidence is limited. Oral mucosa graft urethroplasty is widely used for long and complex adult urethral strictures because of its favorable tissue characteristics. However, its role in pediatric reconstructive urology, particularly in complex post-hypospadias strictures associated with lichen sclerosus (LS), remains rarely reported. We report the case of a 15-year-old patient with severe recurrent anterior urethral stricture after multiple previous hypospadias repairs, complicated by repeated episodes of urinary retention requiring suprapubic catheterization. Histological examination of a urethral biopsy confirmed lichen sclerosus. A single-stage dorsal inlay labial mucosa graft urethroplasty was performed according to the Asopa technique. Intraoperatively, a long-segment anterior urethral stricture extending to the peno-bulbar junction was confirmed. Given the preserved urethral plate width of approximately 2 cm, a dorsal inlay approach was considered feasible. A 7 × 2 cm labial mucosa graft was harvested and quilted to the corporal bed, and the urethra was reconstructed over a 14 Ch Foley catheter with protective dartos flap coverage. The postoperative course was uneventful. At 18-month follow-up, the patient reported satisfactory voiding, no urinary tract infections, no filiform urinary stream, and no clinical evidence of recurrence. This case suggests that dorsal inlay labial mucosa graft urethroplasty may represent a feasible single-stage option in selected adolescents with complex post-hypospadias anterior urethral strictures, particularly when genital skin is unsuitable. Further pediatric experience and longer follow-up are needed to better define indications, durability, and functional outcomes.

## Introduction

Hypospadias is among the most frequent congenital anomalies of the male urethra, and although surgical correction is generally performed in childhood, major complications like urethrocutaneous fistula, meatal stenosis, diverticulum and urethral stricture remain common (1). Strictures after hypospadias repair represent a particularly challenging problem, especially in children and adolescents, where reconstructive strategies are not standardized and evidence is limited (2). Over the last decades, oral mucosa graft urethroplasty has become widely used for long and complex strictures because oral mucosa provides a thick, resilient epithelium, is resistant to infection, and can be easily harvested. As a free graft, its successful incorporation depends on close contact with a well-vascularized recipient bed, meticulous fixation, and avoidance of hematoma or graft movement (3).

Several techniques have been described, including dorsal, ventral, and lateral onlay. The dorsal inlay approach introduced by Asopa in adults urethral strictures allows dorsal graft placement through a ventral sagittal urethrotomy, minimizing urethral mobilization and ensuring a strong graft support (4). While the Asopa technique has been validated in adults with success rates up to 80%–90% (5), the application in pediatric patients is limited. Aldaqadossi et al. (6) reported encouraging outcomes with substitution urethroplasty in children with long anterior urethral strictures, supporting the feasibility of complex reconstructive approaches in selected pediatric patients. However, its use in pediatric reconstructive urology remains rarely reported, especially in complex post-hypospadias strictures associated with lichen sclerosus. We describe a 15-year-old boy with a history of failed hypospadias repair and LS who underwent dorsal inlay labial mucosa graft urethroplasty using the Asopa technique.

## Case presentation

A 15-year-old male presented to our department with severe urethral stricture following previous corrective surgery for hypospadias.

At birth, the patient was diagnosed with distal hypospadias with a subcoronal meatus and associated ventral penile curvature. The primary repair consisted of a two-stage Bracka procedure using a preputial graft. The first stage, performed at 6 months of age, included correction of the ventral curvature and graft placement; the second stage, consisting of tubularization of the grafted urethral plate, was performed 6 months later. The exact degree of the initial ventral curvature was not specified in the operative reports from early childhood.

The patient remained asymptomatic until the age of 7 years, when he developed straining to void and weak urinary stream. Cystoscopy revealed a urethral diverticulum, and a suprapubic cystostomy was placed. One month later, cystography confirmed the presence of a pseudodiverticulum located 18 mm from the external urethral meatus associated with meatal stenosis. Surgical diverticulectomy, urethroplasty and meatoplasty were performed.

At the age of 12 years, a new clinical worsening occurred, with evidence of a thin urinary stream and a pseudodiverticulum located near the penoscrotal junction. Cystoscopy showed a long-segment urethral stricture involving the anterior urethra up to the penoscrotal junction. During the same endoscopic evaluation, a biopsy of the stenotic urethral segment at the level of bulbar urethra was performed. Histological examination of the urethral biopsy confirmed lichen sclerosus. Surgical correction was proposed but declined by the family. Six months later, the patient was hospitalized for hematuria and dysuria and underwent urethral diverticulectomy and urethroplasty.

**Figure 1:**
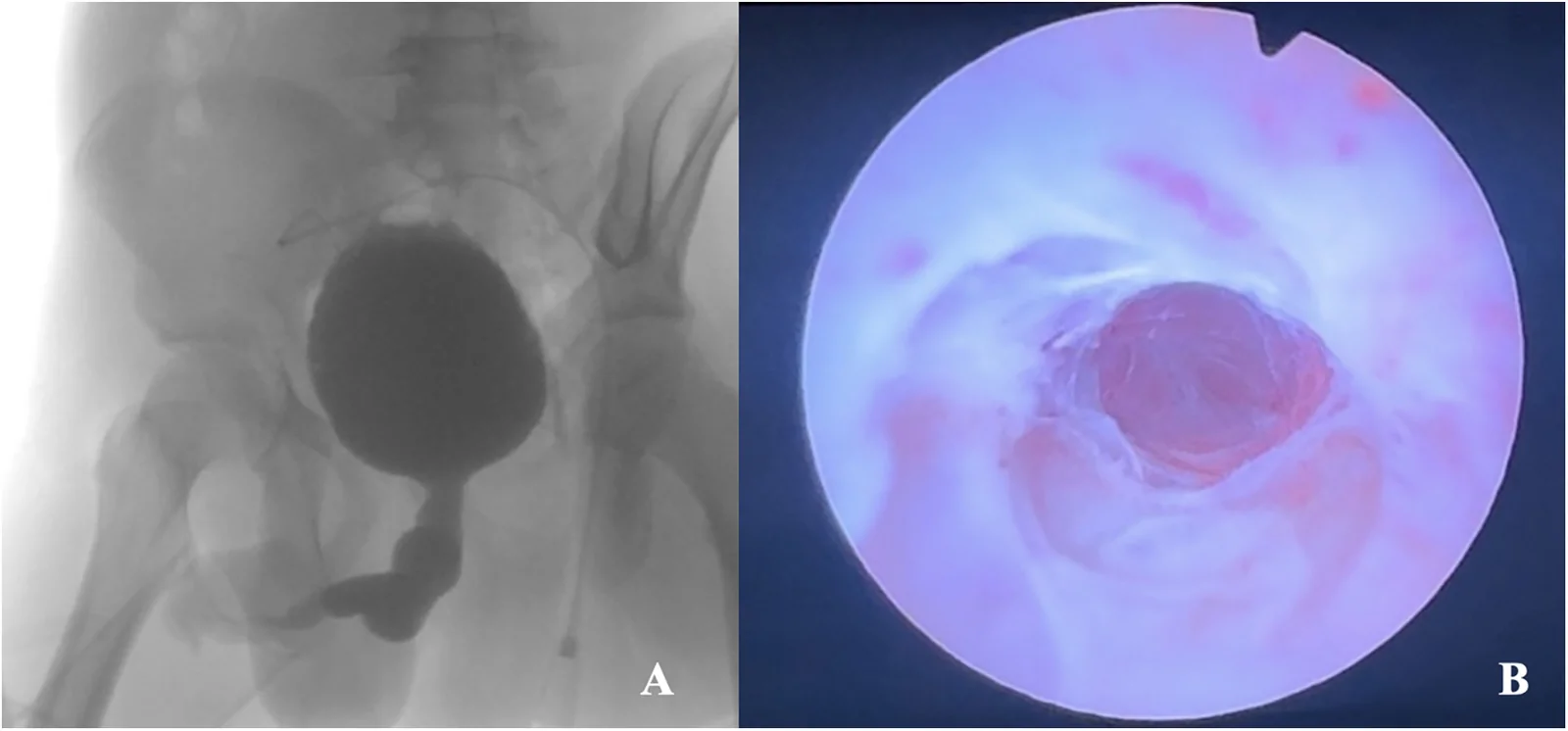
Preoperative findings. **(A)** Voiding cystourethrography showing a stenotic appearance of the penile urethra, with upstream urethral dilatation and a tortuous course. **(B)** Cystourethroscopy showing a long-segment anterior urethral stricture.

After a short symptom-free period, the patient experienced recurrent episodes of urinary retention requiring repeated suprapubic catheterization. Given the progression of symptoms, voiding cystourethrography was performed, showing a stenotic appearance of the penile urethra, with upstream urethral dilatation, tortuous course, and mid-proximal angulations (Figure 1A).

Therefore, the family and patient accepted definitive surgical management. The main clinical events, diagnostic findings, treatments, and outcomes are summarized in Table 1.

**Table 1:** Clinical timeline of the patient.

Timeline	Clinical event	Diagnostic findings	Surgery	Outcome
Birth	Distal penile hypospadias (subcoronal meatus) with ventral curvature	Clinical diagnosis	-	Planned surgical correction
6–12 months	Primary hypospadias repair	-	I - II stages of Bracka using a preputial graft	Initially asymptomatic
7 years	Straining to void, weak urinary stream	Urethral diverticulum; pseudodiverticulum 18 mm from meatus; meatal stenosis	Cystostomy, diverticulectomy, urethroplasty, meatoplasty	Symptom improvement
12 years	Thin urinary stream	Long-segment anterior urethral stricture; cystoscopy with urethral biopsy confirming lichen sclerosus	Surgery proposed but declined	Persistent/recurrent symptoms
12.5 years	Hematuria and dysuria	Recurrent urethral pathology	Diverticulectomy and urethroplasty	Transient improvement
15 years	Complex anterior urethral stricture	5-cm stricture; adequate urethral plate	Asopa dorsal inlay labial mucosal graft urethroplasty	Uneventful postoperative course

**Figure 2:**
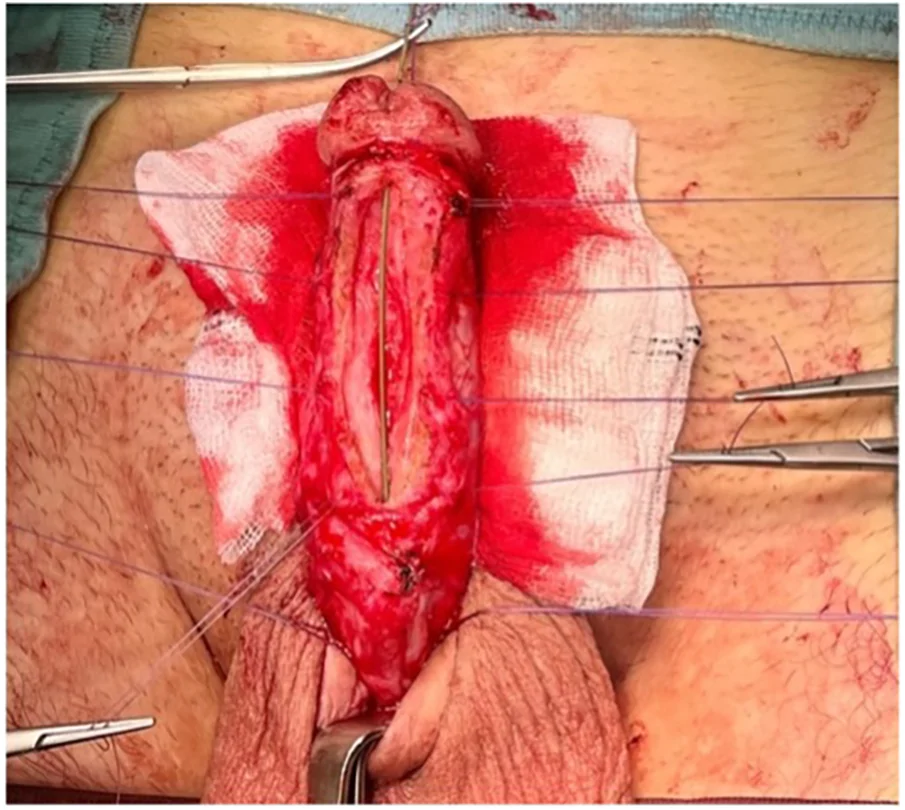
Intraoperative ventral urethrotomy with exposure of the divided urethral plate before dorsal inlay graft placement.

**Figure 3:**
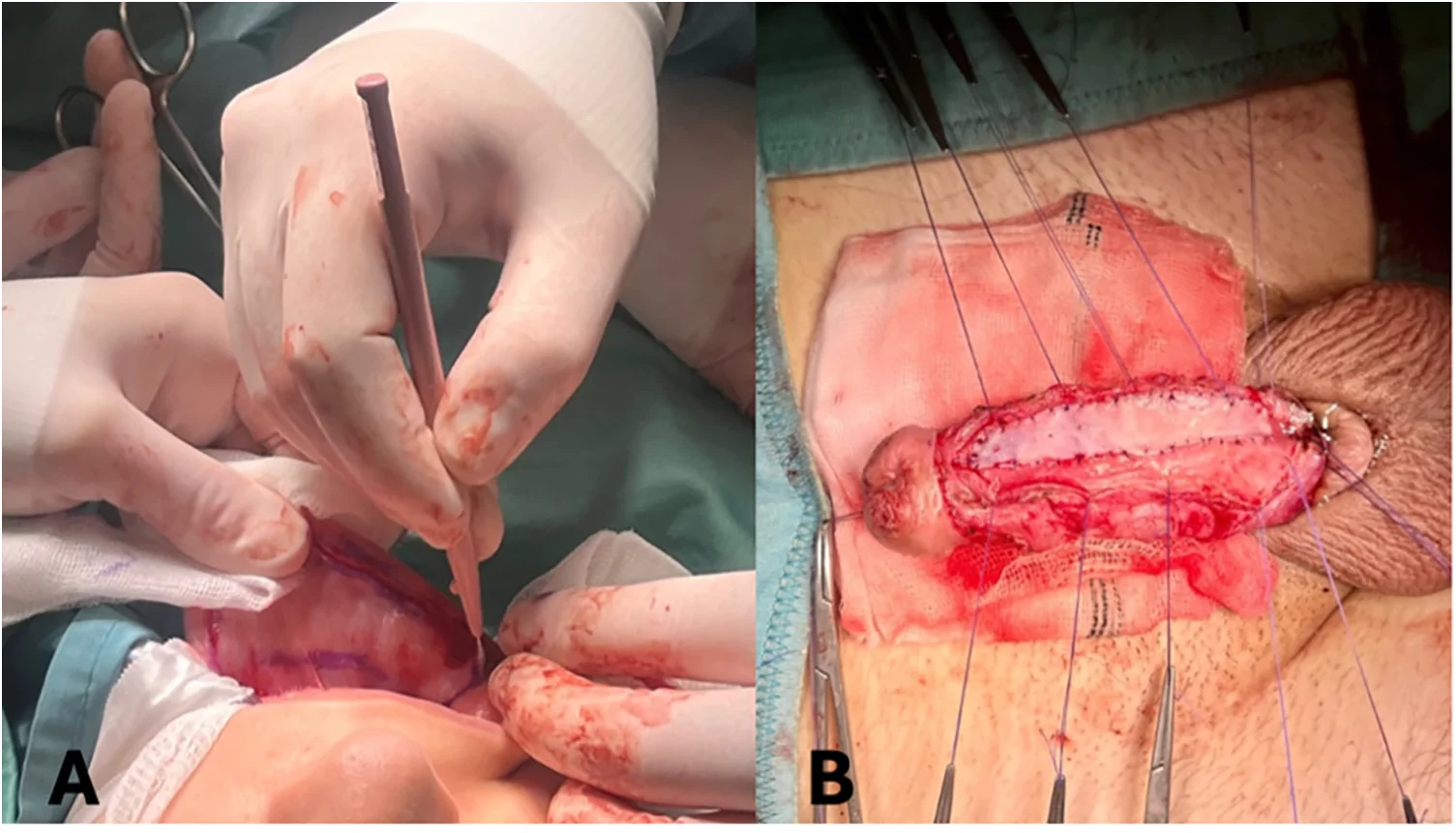
Intraoperative steps of graft reconstruction. **(A)** Labial mucosa graft harvesting; **(B)** Labial mucosa graft quilted dorsally to the corporal bed according to the Asopa technique.

The patient underwent dorsal inlay labial mucosa graft urethroplasty according to the Asopa technique. Cystourethroscopy confirmed a long segment urethral stricture, approximately 5 cm in length, extending to the peno-bulbar junction (Figure 1B). A guidewire was advanced into the bladder. A longitudinal ventral urethrotomy of the penile urethra was carried out up to the penoscrotal junction. Passage of a 16 Ch Foley catheter into the bladder confirmed proximal urethral patency. Both the urethral plate appearance and its width of 2 cm allowed a dorsal inlay approach. A dorsal urethrotomy was carried out (Figure 2), and a 7 × 2 cm labial mucosa graft was obtained from the lower lip and quilted to the corporal bed according to the Asopa technique (Figures 3A,B). This provided adequate dorsal augmentation of the urethral plate (Figure 3B).

The urethra was reconstructed over a 14 Ch Foley catheter with a two-layer closure (5–0 PDS running suture plus 4-0 reinforcing stitches), with a dartos flap protective layer. The postoperative course was uneventful. A urethral Foley catheter and a suprapubic catheter were maintained for urinary drainage and removed after three weeks without complications. At 1-month follow-up, uroflowmetry showed a slightly flattened flow curve, with a voided volume of 320 mL, a maximum flow rate (Qmax) of 12 mL/s, and a post-void residual volume of 20 mL. The 6-month uroflowmetry showed comparable findings, confirming a similar functional pattern without deterioration. Clinically, the patient reported satisfactory voiding, with no urinary tract infections, no filiform urinary stream, and an average voiding interval of approximately 2.5 h. At 18-month follow-up, he remained clinically asymptomatic, with no further episodes of urinary retention, no need for suprapubic catheterization, and no clinical evidence of recurrence. The labial mucosa donor site healed uneventfully, with no bleeding, infection, persistent pain, or limitation of mouth opening.

## Discussion

The management of anterior urethral strictures continues to represent a challenge, especially in the pediatric population and in patients with a history of failed hypospadias repair. Late complications after hypospadias repair may present many years after the initial surgery and include urethrocutaneous fistula, diverticulum, meatal stenosis, recurrent curvature, and urethral stricture (7, 8). The risk and type of complications cannot be attributed to age at surgery alone, but are influenced by several factors, including the severity of the original hypospadias, tissue quality, previous surgical history, surgical technique, surgeon experience, and duration of follow-up (7, 8). In redo cases, repeated operations may further compromise local vascularity and increase scarring, making reconstruction more challenging (7). For these reasons, post-hypospadias strictures should be considered a distinct and complex subgroup of anterior urethral strictures, requiring individualized surgical planning.

Available treatment options include both staged and single-stage reconstructive procedures. In the present case, a single-stage Asopa dorsal inlay repair was preferred over a staged reconstruction after careful intraoperative assessment. The decision was based on the preserved urethral plate width, approximately 2 cm, the feasibility of dorsal augmentation, the need to avoid genital skin in the setting of LS, and the aim of avoiding further multiple procedures in an adolescent who had already undergone several previous treatments.

The Asopa dorsal inlay oral mucosa graft urethroplasty has been recognized as an effective single-stage option in adults, offering the advantages of ventral access with dorsal graft placement, preservation of the urethral plate, minimal mobilization, and stable graft support on the corporal bed (4, 5, 9). Long-term success rates of 70%–90% have been reported in adults (10, 11), but its application in children remains poorly documented. Aldaqadossi et al. reported encouraging outcomes in children undergoing substitution urethroplasty for long anterior urethral strictures, although pediatric data remain limited (6). Our case suggests that the dorsal inlay approach with oral mucosa graft can be successfully applied in selected adolescent patients with complex post-hypospadias strictures, even in the challenging setting of histologically confirmed LS. Oral mucosa grafts are free grafts rather than vascularized flaps. Their successful incorporation depends on close contact with a well-vascularized recipient bed, meticulous graft fixation, and avoidance of hematoma or graft movement. In the Asopa technique, dorsal placement on the corporal bed provides a stable and well-vascularized support for graft take (4, 5). In the present case, the labial mucosa graft was quilted dorsally to the corporal bed, providing adequate dorsal augmentation of the urethral plate.

In adult practice, several substitution urethroplasty techniques have been established. The dorsal onlay oral mucosal graft urethroplasty described by Barbagli allows the graft to be placed on a well-vascularized recipient bed, but requires circumferential mobilization of the urethra, which may compromise vascular integrity (9). The ventral onlay approach, while technically less demanding and requiring limited dissection, has been associated with a higher risk of sacculation or diverticulum formation, particularly outside the bulbar urethra (9). Cutaneous flaps, including penile or preputial skin, have been employed historically but are currently discouraged because of variable tissue quality and unfavorable long-term outcomes, especially in the presence of LS (2, 12). Excision and primary anastomosis remains the standard for short bulbar strictures but is contraindicated in long or recurrent cases due to the risk of penile shortening and chordee. Within these reconstructive options, the Asopa dorsal inlay urethroplasty integrates the benefits of dorsal graft placement with limited urethral mobilization, offering a balanced, single-stage option that has been reported to achieve outcomes consistent with those of other well-validated approaches (13, 14).

Despite the extensive literature in adults, data in pediatric patients are very limited, especially in the context of strictures following hypospadias repair. Pediatric urethral strictures are rare, with heterogeneous etiologies, most commonly iatrogenic or traumatic, as highlighted in a recent systematic review by Vetterlein et al. (2). Reports of augmentation urethroplasty with oral or labial mucosa in children remain limited; Aldaqadossi et al. described encouraging results, but data remain too limited to draw definitive conclusions (6). Our case adds to this small body of evidence and suggests that the Asopa dorsal inlay technique may be selectively extended to adolescent reconstructive surgery.

The concomitant presence of LS further increases the complexity of urethral reconstruction, as it is associated with progressive fibrosis and high recurrence when genital skin is reused (12). In these cases, oral mucosa is the graft of choice. Recent studies comparing Asopa and Kulkarni techniques in adult LS strictures have shown equivalent outcomes, confirming the reliability of oral mucosa in this setting (14). Our case, with histologically proven LS, highlights the suitability of this approach even in pediatrics.

The main limitations of this report are its single-case design, the relatively limited follow-up, the absence of initial photographic documentation from infancy, and the absence of postoperative urethrographic assessment. Although preoperative voiding cystourethrography documented the urethral abnormality, postoperative evaluation was based on clinical assessment and uroflowmetry. Therefore, no definitive conclusions regarding long-term durability can be drawn. Nevertheless, this case may support consideration of the Asopa dorsal inlay technique in carefully selected adolescent patients with complex post-hypospadias strictures.

## Conclusions

The Asopa dorsal inlay urethroplasty is a well-established reconstructive technique in adult anterior urethral stricture disease, offering advantages such as partial urethral mobilization, preservation of vascular supply and the possibility to use a single-stage procedure. To date, most reports have focused on adult patients, while pediatric experience with labial mucosa graft urethroplasty for complex post-hypospadias strictures remains limited. This case suggests that the Asopa dorsal inlay labial mucosa graft urethroplasty may be considered in selected adolescents with complex anterior urethral strictures after failed hypospadias repair, particularly when genital skin is unsuitable because of lichen sclerosus. Further pediatric experience with longer follow-up is needed to better define indications, durability, and functional outcomes.

## Patient perspective

The patient and his legal guardians reported satisfaction with the postoperative outcome, particularly regarding the restoration of spontaneous voiding and improvement in urinary stream. They expressed relief at avoiding further episodes of urinary retention and additional suprapubic catheterization. Written informed consent for the anonymized publication of this case report and associated clinical images was obtained from the patient's legal guardians; patient assent was also obtained when appropriate.

## Data Availability

The original contributions presented in the study are included in the article/Supplementary Material, further inquiries can be directed to the corresponding author.
